# Towards universal health care coverage in low- and middle- income countries: integrating refugees into national health systems

**DOI:** 10.1186/s13031-025-00684-y

**Published:** 2025-07-15

**Authors:** Fadi El-Jardali, Sara Bennett, Paul Spiegel

**Affiliations:** 1 Department of Health Management and Policy, Faculty of Health Sciences, American University of Beirut, Beirut, Lebanon; 2 Knowledge to Policy (K2P) Center, American University of Beirut, Beirut, Lebanon; 3 Center for Systematic Reviews on Health Policy and Systems Research (SPARK), American University of Beirut, Beirut, Lebanon; 4 Department of Health Research Methods, Evidence, and Impact (HEI), McMaster University, Hamilton, ON Canada; 5 Johns Hopkins Bloomberg School of Public Health, Baltimore, MD USA; 6 Johns Hopkins Center for Humanitarian Health, Baltimore, MD USA

**Keywords:** Refugees, Universal health care, Health system, Low- and middle-income countries

## Introduction

Globally, the number of people forcibly displaced has reached an unprecedented record surpassing 100 million, including 26.3 million refugees, in May 2022 [1]. The vast majority of the world’s refugees (83%) are hosted by and living outside of camps amongst national populations in low- and middle-income countries (LMICs) [2]; often adjacent to the countries experiencing crises or conflict. Many of these host countries are themselves already experiencing political instability and limited resources. This, in turn, hinders their ability to provide adequate healthcare access - a fundamental human right - to refugees and nationals alike. The situation is further complicated by the protracted nature of current refugee crises and the tremendous health needs of refugee populations encompassing communicable and non-communicable diseases, notably mental health conditions, and injuries including those linked to gender-based violence (GBV).

As many governments embark on health reforms towards achieving the Sustainable Development Goals, in particular universal health coverage (UHC), there is a growing need for sustainable and comprehensive approaches that can provide adequate financial risk protection and access to quality essential health care for all. Historically health services for refugees have often been provided through separate, parallel systems, particularly in refugee camps, but there is increasing recognition that such a model is unsustainable and leads to missed opportunities to benefit both refugee and host populations. As described in one of the papers in this supplement (Elnakib et al.) the integration of refugees within national health systems has emerged as a major element of humanitarian policies set out in recent the United High Commissioner for Refugees (UNHCR) and World Bank policy statements [3, 4], and sometimes referred to as the “humanitarian-development nexus”. More recently the World Health Organization (WHO) global action plan 2019–2023 on promoting the health of refugees and migrants explicitly notes the need to strengthen the provision of a variety of health services for refugees as part of UHC and “leaving no one behind” [5].

During all phases of an emergency, but particularly during the post-emergency phase of a crisis, integrating refugees into health systems offers a number of benefits. For example, it has the potential to: (1) provide more quality health services more efficiently; (2) bolster weak national health systems through the infusion of additional material, financial, human resources and technical support; (3) drive the development of services that may not exist (e.g. mental health, gender-based Violence); (4) support increased understanding and reduce stigma and discrimination between refugee and host populations; and (5) address health, socioeconomic and other inequities between these populations [6–8]. The issue of integrating refugees into health systems, however, still faces resistance from some governments and host populations who may be concerned about the effects of integration on already stretched health systems, longer term financial costs that may ultimately fall to the host governments, as well as the expectations concerning broader societal integration and acceptance, that health system integration may give rise to.

## Overview of the supplement

To-date there has been relatively limited research exploring the policies, models and implications of integrating refugees into health systems in LMICs [6–9]. Notably, there is limited evidence available to international, regional and national actors in terms of how policies integrating refugees have been implemented and received by local stakeholders, the types of arrangements that may work best in particular contexts, or the evidence base available to evaluate such strategies. In this supplement, we explore issues related to the question of integration by focusing on three countries, Lebanon, Jordan, and Uganda, hosting large refugee populations. Our analyses focus in particular on integration of Syrian refugees who fled Syria after the escalation of the civil war in 2012, and on South Sudanese refugees who fled to Uganda around 2013/14.

While all three countries involved in the study have integrated refugees to some degree, they represent very different models for doing so. Specifically, the health system in Lebanon is market-oriented, with a strong private sector. Given this, and financial challenges faced by the government, refugee integration into the health system has emerged largely through external funding flowing through non-government entities to purchase services from both public and private health care providers. In Jordan, there is a far greater reliance on public health care providers which are largely tax financed through the civil insurance program [10]. In this context, external funding agencies such as UNHCR have made funds available to the government who in turn reimburse public health care providers for services offered to refugees. Finally, in Uganda, where there has been a very long-standing public commitment to refugee integration, districts which host large numbers of refugees approach health service planning for both refugee and host populations in an integrated fashion. While additional funding for refugees is received from external donors by the government, refugee and host communities in affected districts have identical entitlements to services provided through the local health care system.

Study teams in each of these three countries sought to understand how policies integrating refugees have evolved, particularly since 2012 the start of the Syrian refugee crisis in Jordan and Lebanon, and the South Sudanese crisis in Uganda, and how these policies have been implemented in practice. The teams employed a mix of qualitative and quantitative methods including (i) document review in order to understand the evolving policy environment; (ii) key informant interviews with policymakers, health service managers, health care providers, and civil society organizations (including those representing refugee populations); and (iii) review of routine data sources such as data from health management information systems, and records held by international organizations such as UNHCR.

This supplement is composed of six papers in addition to this editorial. The first paper (El Nakib et al.) provides a global level perspective on how policy positions regarding the integration of refugees into health systems evolved, addressing questions such as the power of different actors, the policy networks that supported change, the framing of arguments, and critically the broader policy context, notably the impact of the Syrian refugee crisis on the position of European governments. The next three papers explore similar questions about how policies on refugee integration into health systems got onto the national agendas in Lebanon (El-Jardali et al.), Jordan (El Nakib et al. a) and Uganda (Komakech et al.) and how those policies have been implemented in practice. These three papers offer both contrasts and similarities. In Uganda, in the context of strong political commitment to refugee integration for decades, there has been a sustained and steady process of policy change, learning and further institutionalizing policies so that guidance on refugee integration is fully captured across policy and guidance documents down to the local level. By contrast, refugee integration in Jordan and Lebanon has been a more recent phenomenon, with some wavering of government commitment over time. In particular, commitment to refugee integration has been closely aligned with the degree of support from international partners such as UNHCR and the World Bank, and occasional relapses in government commitment when financial implications of integration have appeared prohibitive. While similar study tools were used across the three countries, the stories of policy change matched differing policy theories: the Lebanon and Jordan papers employ Kingdon’s three stream model [11] of policy change reflecting the opening of a policy window shortly after the Syrian refugee crisis occurred; the Uganda paper uses the Walt and Gilson policy triangle model [12] to reflect the more incremental processes through which policy change occurred; and the global paper uses Shiffman and Smith’s framework for policy priority [13].

Two additional papers are included in this supplement. One is a more in-depth case example from Lebanon on the implications of refugee integration policies on health systems (e.g. access, availability and quality). This paper captures the perceptions and experiences of stakeholders including host and refugee populations towards refugees’ integration. A final paper reflects on the status of routine health information systems across all three study countries, and the degree to which they support understanding of the needs of refugee populations; the services that this population are using; and the impact of refugee integration on the broader health system. The paper makes clear that health information systems for refugee and host populations typically remain siloed creating risks of missing data, as well as duplication of data. Further, the inability of health information systems to disaggregate refugee from host populations limits opportunities for understanding health service access issues in both populations, and developing targeted interventions to address them.

## Policy implications and future research

The three countries included in the papers in this supplement were selected in part for their differing approaches to integrating refugees into health systems, however the research has demonstrated how integration strategies need to be adapted to consider both different societal attitudes towards refugees, as well as differences in the organizational structure and financial arrangements of health systems. The integration of refugees into health systems cannot be a single homogenous strategy, but rather an approach that requires careful, context-specific fine tuning according to existing health systems, donors’ policies and commitments, and evolving socioeconomic and political contexts in the hosting countries and the region. While the three countries reported on here reflect different modalities for integration, the global community may benefit from having a series of alternative models that vary according to diverse contexts and factors mentioned above.

One aspect of the health system that is particularly significant when considering approaches to integrating refugees is the extent to which host country governments have strong commitments to UHC for their own population. As the paper by El-Jardali et al. describes the lack of commitment to UHC in Lebanon has adverse implications for both refugee and host populations, especially since the economic collapse, as both these populations now face significant financial barriers in accessing care. Framing the refugee integration agenda as part of broader commitments to UHC for all populations is essential.

Experience in both Jordan and Lebanon underscores the critical role that external funding can play in incentivizing and sustaining the integration of refugees into health systems. In particular, the 2014 and 2018 policy changes in Jordan that required refugees to pay increasingly high charges for using health care were only repealed once World Bank loans, as well as the establishment of a multi-donor trust fund, reassured the government of external support. While all parties seem to agree that integrating refugees into health systems makes economic sense and offers new opportunities for health systems strengthening, there is also a real risk that once the refugee crisis has faded from the headlines, the host countries, who are often not high-income countries, will be left with the financial burden. Given the long-term nature of refugee crises, financial commitments from international development banks and high-income country donors also need to be long term in nature.

Beyond health care financing, this supplement also provides policy implications concerning information systems and the health workforce. As the paper by Bou-Karroum et al. describes, typically country health information systems are not adequately prepared to capture the implications of refugee influxes; the refugee population may be added into the denominator of indicators on an occasional basis leading to large discrepancies in indicator trends, and most health information systems do not differentiate between refugee and non-refugee populations. Consequently, to track refugee integration policies better in the future, much more needs to be done to support a more holistic and integrated health information system with clearer guidance about how to integrate refugees (as well as other types of migrants) into information systems\. Finally, in both Jordan and Lebanon, Syrian health care workers are prohibited from providing health care services. While, in general, granting employment authorization to refugees can be politically sensitive, there is surely a strong argument for providing exceptional permits for scarce and sorely needed professions such as health care workers.

## Conclusion

This journal supplement begins to address a critical knowledge gap on the issue of integrating refugees in national health systems. The papers presented here address key elements for successful policies on integration such as political commitment, sustainable financing sources, strong primary health care systems, refugee data integration in national health information systems, and multi-sectoral collaboration and coordination among different actors. While we hope that the evidence will empower decision-makers within the three countries to learn from and consider alternative approaches to refugee integration, as well as strengthening the design of health reform strategies, it is clear that this question remains under-researched, and poorly understood. More studies that explore the operational details of how refugee integration can best be financed, managed and evaluated, are needed.

## Data Availability

Not applicable.

## Abbreviations

LMICs: Low- and middle-income countries
UHC: Universal health coverage
UNHCR: United High Commissioner for Refugees
WHO: World Health Organization
